# An Alternative Technique for Transumbilical Single-Port Laparoscopic Percutaneous Precise Closure of the Inguinal Hernia Sac in Children: A 3-Year Single-Centre Study

**DOI:** 10.1155/2021/6679519

**Published:** 2021-06-25

**Authors:** Xiaoliang Xu, Guojian Ding, Xuefeng Cao, Tingliang Fu, Fengchun Cheng, Shuai Sun, Lei Geng

**Affiliations:** ^1^Department of Pediatric Surgery, Binzhou Medical University Hospital, Binzhou, Shandong Province, China; ^2^Department of Hepatobiliary Surgery, Binzhou Medical University Hospital, Binzhou, Shandong Province, China

## Abstract

**Objective:**

To evaluate the safety and reliability of a novel technique of single-port laparoscopic-assisted percutaneous precise closure of the inguinal hernia sac in children.

**Methods:**

From September 2016 through September 2019, children with inguinal hernia(s) treated with single-port laparoscopic-assisted percutaneous extraperitoneal closure using a guide wire were enrolled in this study. Operative time, surgical complications, recurrence rate, and cosmetic results were collected.

**Results:**

A total of 917 cases with inguinal hernia(s) were collected. Among them, there were 886 (96.61%) boys and 31 girls. Their mean age was 5.2 ± 3.7 years. There were 693 (75.57%) cases with unilateral hernia. There were 224 cases with bilateral hernias or patent processus vaginalis, including 135 (14.72%) cases with an open contralateral ring which was confirmed intraoperatively. Twenty-three (2.51%) needed another port to complete the hernia sac separation. The operation time was 24.7 ± 5.2 min and 14.6 ± 3.8 min in bilateral and unilateral ones. Three cases complained of numbness in the thigh region or groin pain which subsided without medication in the 2^nd^ postoperative month. There was no recurrence, and the incision scars were nearly invisible.

**Conclusion:**

Single-port laparoscopic-assisted percutaneous extraperitoneal closure using a guide wire is a safe, less cost, and reliable technique in the treatment of inguinal hernia in children.

## 1. Introduction

Indirect inguinal hernia is one of the most common diseases in the pediatric age group. Indirect inguinal hernias, hydrocele, or a combination of both are usually related to the persistent patency of the processus vaginalis without an obvious abdominal wall defect. Laparoscopic-assisted high ligation of the inguinal hernial sac has been widely used in children [1]. Compared with open hernia repair, laparoscopic hernia repair has the advantages of rapid postoperative recovery, excellent cosmetic appearance, and fewer complications [2]. Moreover, the contralateral hidden hernia can be found and repaired simultaneously [3]. Other advantages include without dissecting the inguinal canal, less risk of spermatic cord and vas deferens damage, less iatrogenic cryptorchidism, and testicular atrophy [4]. There are a variety of surgical approaches for laparoscopic hernia repair, such as extraperitoneal or transabdominal paths, which usually need 2 to 3 ports. Single-port technique has been increasingly used in extracorporeal ligation of the hernia sac in children with indirect inguinal hernia [3–7].

However, there are several disadvantages of these surgical approaches, such as more abdominal wall tissue ligation and forming angle of the spermatic cord due to traction. Extracorporeal knot-tying and burying the knots subcutaneously may give rise to tissue reactions, stitch-related sinus, chronic infection, or granulomas [8]. And the inclusion of muscular tissue within the knots may lead to an increased recurrence rate due to the loosed knots through cutting the abdominal wall tissues [8]. Li et al. [9] introduced double hooked hernia needle and its use to avoid the ligation of abdominal wall tissues and to reduce the recurrence rate, but the cost may increase. Hereby, we describe a modified technique using a guide wire to realize the precise high ligation of the inguinal hernia ring with good outcome and less cost.

## 2. Materials and Methods

### 2.1. Patient Selection, Measurements, and Preparation

From September 2016 through September 2019, a total of 917 pediatric patients with indirect inguinal hernia in our hospital were enrolled in this study under informed consent. Inclusion criterion is as follows: all children with indirect inguinal hernia (unilateral or bilateral, recurrent, and unilateral with questionable contralateral hernia) who underwent transumbilical single-port laparoscopic-assisted hernia sac ligation during the period of study. Exclusion criterion is as follows: patients with other severe conditions who did not tolerate laparoscopic surgery.

### 2.2. Surgical Procedure

**Figure 1:**
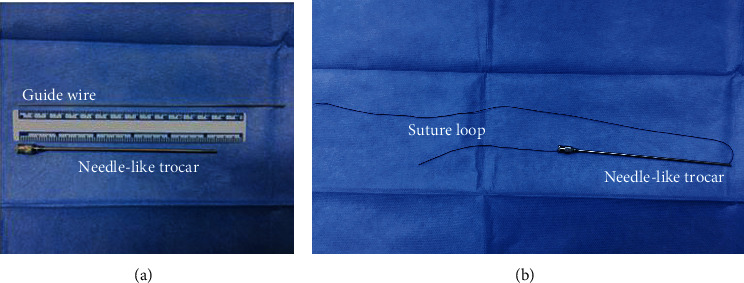
(a) The self-made needle-like trocar and guide wire; (b) needle-like trocar with suture.

A 5 mm umbilical trocar was used for insufflation and insertion of a 30° laparoscope. Self-made needle-like trocar (diameter 1.8 mm, length about 135 mm), guide wire (retrofitted by Kirschner wire, diameter 1 mm, length 180 mm)(Figure 1(a)), and 3-0/2-0 nonabsorbable silk sutures were used for the surgical procedure. The suture was threaded inside the barrel, and the tail pulled back (Figure 1(b)).

**Figure 2:**
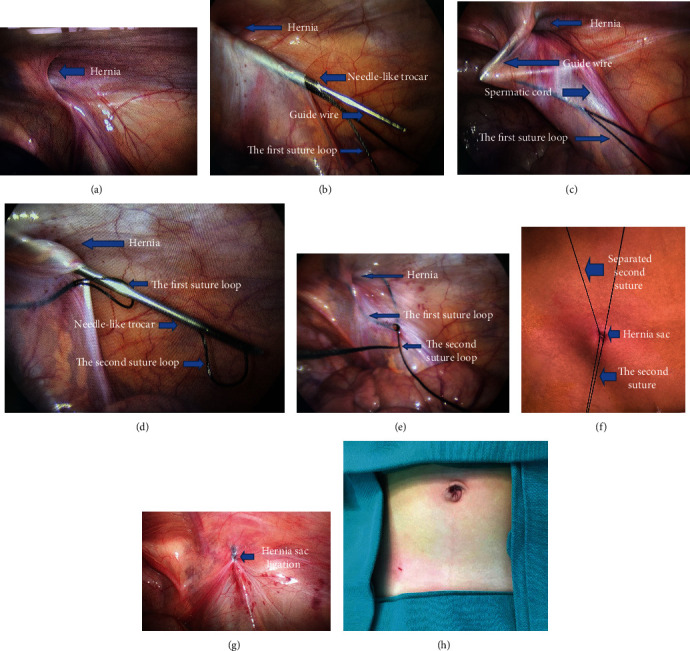
(a–h) Modification of the technique with single-port laparoscopic assisted hernia sac ligation extraperitoneally. (a) Confirming the diagnosis by laparoscopic exploration. (b) The guide wire is inserted into the trocar and stayed it in the cannular while placing the first suture loop in the abdominal cavity and withdrawing the needle-like trocar to insert the second suture loop. (c) The integrity of the hernia sac was reassessed. (d) The second suture loop is pulled into the peritoneal cavity and entered the first suture loop. (e) The second suture loop is locked inside the loop for retrieving the suture. (f) Sutures are tied separately. (g) The ligated suture knots are located in the extraperitoneal space. (h) Suturing incision located in the umbilicus.

The surgical techniques are as follows. The patient was placed supine in the Trendelenburg position at 15-20° with inclining on the contralateral side at about 20°. General anesthesia with laryngeal mask was used in all patients. A 5 mm vertical transumbilical skin incision was created within the umbilical cicatrix. The peritoneal cavity was exposed. A 5 mm port was inserted into the abdomen cavity. A 30° laparoscope was introduced. The pressure of pneumoperitoneum of carbon dioxide was maintained from 8 to 10 mmHg. The indirect inguinal hernias were confirmed by laparoscopic exploration (Figure 2(a)), and the contralateral side was inspected accordingly.

A 2 mm skin incision was then made between the lateral and medial borders of the open internal ring. After dissecting the superficial tissues, a self-made, straight fine-tipped needle-like trocar with a 3-0 synthetic monofilament, nonabsorbable suture was inserted through the 2 mm skin incision and delicately advanced toward the extraperitoneal space on the medial side of the internal ring. When it was over the vas deferens and spermatic vessels in males or under the round ligament in females, the trocar was entered the abdominal cavity through the peritoneum and the suture was pulled through the barrel of the trocar assisted with the laparoscope, creating the first internal suture loop (about 40 mm in length) (Figure 2(b)). If it was difficult to separate the peritoneum from the vas deferens, the hydrodissection technique [9] could be used by injecting aseptic normal saline solution into the retroperitoneal space through the needle-like trocar. Then, a guide wire was inserted into the needle-like trocar before withdrawing it outside for the second insertion (Figure 2(b)).

The integrity of the peritoneum around the hernia sac was inspected while the guide wire was pulled back into the extraperitoneal space (Figure 2(c)). Then, needle-like trocar with the second suture was reinserted through the guide wire into the extraperitoneal space. This made the needle-like trocar insertion into the extraperitoneal space in the first suture's channel. The extraperitoneum of the lateral part of the internal ring was separated, and the needle-like trocar entered the abdominal cavity through the same hole of the first suture placement. The first suture loop was passed through the needle-like trocar, and the second suture loop was pulled into the peritoneal cavity assisted with the laparoscope and stayed the second suture loop (about 6 cm in length) in the abdominal cavity (Figure 2(d)). Withdrawing the needle-like trocar, the second suture loop was tightly locked by the first suture loop (Figure 2(e)).

Pulling the first suture loop outside of the abdominal wall, the second suture loop was brought out of the abdominal wall simultaneously. Then, the second suture loop was cut to form two sutures (equal in length). Pulling the suture tip to make sure it was the same suture, then the neck of the hernia sac (internal ring) was ligated extrocorporeally (Figure 2(f)). While the suture was tied, the pneumoperitoneum and gas in the distal part of the hernia sac should be expelled through the trocar in the umbilicus region. The suture knots were precisely located in the extraperitoneal space (Figure 2(g)).

The umbilical incision was closed with an absorbable suture (Figure 2(h)), and the small incision in the inguinal area did not need suture. It can be sealed with self-made steri-strip, not shown in Figure 2(g).

### 2.3. Postoperative Management

Liquid food was permitted beyond 2 hours of observation, according to the patients' condition. The patients were discharged from the hospital on the 2^nd^ or 3^rd^ postoperative day at our institution. All patients were instructed to follow up in the clinic or telephone interview at one week, one month, and six months after operation.

The data of operative time, recurrence rate, surgical complications, and cosmetic results were collected.

## 3. Results

A total of 917 cases with inguinal hernia(s) were collected. Among them, there were 886 (96.61%) boys and 31 girls. Their mean age was 5.2 ± 3.7 years (ranged from 10 to 132 months). There were 693 (75.57%) cases with unilateral hernia, including 490 (70.71%) cases in the right side and 203 in the left side. There were 224 cases with bilateral hernias or patent processus vaginalis, preoperative diagnosis was made in 89 cases, and the other 135 (14.72%) cases with an open contralateral internal ring confirmed intraoperatively were made the diagnosis. All cases did not receive antibiotic prophylaxis during the perioperative period. No urethral catheter insertion was needed during the operation.

Hernia repairs by single-port laparoscopic-assisted procedure were successfully performed on 894 cases. Twenty-three (2.51%) cases needed to insert another trocar (3 mm in diameter) to repair the hernia due to the large scars around the internal ring in 15 cases and sliding inguinal hernia in 8 cases. The mean operative time was 14.6 ± 3.8 minutes (range = 8‐27 min) for cases with unilateral hernia and 24.7 ± 5.2 minutes (range = 15‐34 min) for bilateral. Intraoperative blood loss was minimal. The mean length of hospital stay was 2.4 ± 0.7 days after operation. During the follow-up period, three cases had complained of numbness in the thigh or groin pain which subsided without medication in the 2^nd^ postoperative month. No spermatic cord damage, scrotal hematoma, suture granuloma or sinus infection, iatrogenic cryptorchidism, or testicular atrophy occurred. The follow-up period was at least 6 months. There was no recurrence, and the scars were nearly invisible.

## 4. Discussion

In children, inguinal hernia is one of the most common surgical conditions [1–8]. Compared with open surgery, the procedure of laparoscopic-assisted inguinal hernia repair has several advantages, including clearer visualization of the anatomy, less risk of vas deferens and vessel injury, ligation of the neck of the hernia sac without additional dissection, minimally invasive, less postoperative pain, and good cosmetic outcome [2, 10]. Moreover, contralateral patent processus vaginalis can also be identified and treated simultaneously using the same method [6, 8, 10]. It is also suitable for recurrent hernia repair as described by Lee and Park [11]. There are a variety of laparoscopic repair procedures for the management of indirect inguinal hernia in children. For example, compared with intracorporeal suturing [12], the technique of extracorporeal ligation [8, 9] usually has some advantages, including shorter operative time, lower recurrence rate, and better cosmetic results. However, the previously described techniques of extracorporeal ligation have common drawback, i.e., knots are often located subcutaneously. Because self-made needle-like trocar needs to enter the extraperitoneal space twice, it was hard to make the needle-like trocar insertion to the extraperitoneal space through the same channel. This may cause infection, chronic granuloma, or suture knot-related sinus. Moreover, due to the inclusion of muscular tissues in the suture and cutting through these tissues gradually, the suture may loosen and lead to recurrence [8].

Although the technique of laparoscopic hernia repair has about 20 years history, different techniques have been described with no consensus on the standard technique [10, 13, 14]. Li et al. [9] described an inner two-hooked cannula with a nonabsorbable suture insertion into the corresponding point of the internal ring, which was kept in an identical subcutaneous path. The internal ring of the hernia can be ligated extraperitoneally. This technique can avoid to ligate redundant tissues, but the cost increases accordingly. We used the guide wire to ensure the second insertion of the needle-like trocar in the same channel. Furthermore, while withdrawing the guide wire, the integrity of the hernia sac, the vas deferens, and spermatic vessels can be reassessed. Extracorporeal knotting can easily be conducted, and the suture knots slide into the real extraperitoneal space rather than buried in the subcutaneous tissue. From our experience, the procedures were proved to be more precise and easier to learn with less cost. More importantly, it can avoid the occurrence of the sinus formation related to suture knots and decrease the recurrence rate. Hydrodissection technique makes the separation of the vas deferens and spermatic vessels from the peritoneum easier and safer [9]. Occasionally, if the suture did not insert through the hernia sac completely, it needed to reinsert the needle-like trocar to introduce the suture loop into the peritoneal cavity, especially in cases with a scarred or giant hernia sac. Adding another 3 mm trocar to assist the separation may be a choice.

In addition, while pushing the suture loop into the abdominal cavity, surgeons must observe the tip of the needle-like trocar in the field of laparoscopy to avoid bowel and vessel injury. In our series, there were three children who complained of transient numbness or groin pain, indicating to keep the triangle of pain intact is important to reduce the risk of occurring this symptom postoperatively, although it occurred rarely [11].

Good cosmetic outcomes were achieved by single-port laparoscopic-assisted percutaneous extraperitoneal closure using a guide wire. Long-term follow-up was needed to assess the reliability of this novel technique in the management of indirect inguinal hernia in children.

In conclusion, this modified technique made the insertion of the second suture in the first suture channel and precisely extraperitoneal ligation of the hernia sac could be easily conducted. It is reliable with less cost for the management of indirect inguinal hernia in children.

## Data Availability

The data sets generated and analyzed during the present study are available from the corresponding author on reasonable request.
